# Analysis of discrepancies in hemorrhagic transformation and infarct volume in ischemic stroke patients undergoing endovascular treatment

**DOI:** 10.3389/fneur.2026.1783768

**Published:** 2026-03-06

**Authors:** Jianqiang Hu, Shuyu Ma, Jiawei Zhang, Kefangyuan Zheng, Mingqing Cheng, Xin Miao, Jiarui Bao, Donghua Xian, Yalan Fang, Jin Zhang

**Affiliations:** 1 Department of Neurology, Second Hospital of Shanxi Medical University, Taiyuan, Shanxi, China; 2 Clinical College, Shanxi Medical University, Taiyuan, Shanxi, China; 3 Department of Neurology, First Hospital of Shanxi Medical University, Taiyuan, Shanxi, China

**Keywords:** endovascular treatment, hemorrhagic transformation, infarct volume, ischemic stroke, neuralfunctional outcomes

## Abstract

**Objective:**

After endovascular treatment (EVT) for ischemic stroke (IS), clinical observations have shown that some patients with small infarct volumes develop hemorrhagic transformation (HT), while some patients with large infarct volumes do not. This study aims to analyze the factors contributing to these differences and to assess the impact of HT on neurological outcomes.

**Methods:**

A total of 732 patients were divided into small infarct volume (0–15 mL) and large infarct volume (≥70 mL) groups. The incidence of HT, risk factors, neurological outcomes (NIHSS changes), early neurological deterioration (END), and 7- and 90-day mortality rates were compared.

**Results:**

In patients with small infarcts, higher systolic blood pressure, lower triglyceride levels, the number of EVTs, and other factors were related to an increased risk of HT. By contrast, in those with large infarcts, where HT occurred more frequently and was more severe, it was associated with more severe neurological deficits before treatment, elevated albumin levels, and the number of EVTs. PH2 hemorrhage was linked to more severe neurological deficits, higher END rates, and increased short- and long-term mortality, particularly in large infarcts.

**Conclusion:**

Infarct volume is closely related to the occurrence and severity of HT. HT in small infarcts is influenced by hemodynamic and metabolic factors, while in large infarcts, it is linked to extensive brain tissue damage. PH2 hemorrhage is the most adverse prognostic subtype, highlighting the need for careful monitoring and intervention in patients with large infarcts.

## Introduction

Each year, approximately 12 million new stroke cases occur globally, with ischemic stroke (IS) accounting for 62% of these cases. IS remains one of the leading causes of disability and death worldwide (1, 2). With the rapid advancement of endovascular treatment (EVT), an increasing number of patients with large vessel occlusion strokes can now receive reperfusion treatment (3, 4). However, hemorrhagic transformation (HT) remains a common complication after EVT and has been reported in a substantial proportion of patients across different cohorts (5, 6). Severe HT, in particular, has been shown to significantly increase the risk of death and disability, becoming a key factor influencing the efficacy of EVT (7, 8).

Previous studies have suggested that HT occurrence is associated with various clinical factors, including age, blood pressure levels, post-recanalization blood flow velocity, the degree of neurological impairment upon admission and others (9–11). Among these, a larger infarct volume (IV) is considered one of the most important risk factors (12). However, clinical observations reveal that some patients with small infarcts still experience HT, while some with large infarcts do not, indicating that the mechanism behind HT may not be solely dependent on infarct size (13).

To date, systematic research on the relationship between infarct volume and HT remains limited, particularly within the EVT patient population. While larger infarct volumes have consistently been associated with higher rates of HT and worse outcomes, the extent to which risk factors and prognostic impact differ across distinct infarct volume strata remains uncertain. Therefore, this study aims to retrospectively analyze stroke patients after EVT, investigating the factors associated with the occurrence of HT in relation to different infarct volumes. It will also assess the independence of HT risk factors across varying infarct volumes and further compare the impact of HT on short-term neurological outcomes and 90-day mortality in patients with different infarct sizes.

## Methods

### Study population

This study was approved by the Ethics Committee of the First Hospital of Shanxi Medical University. We retrospectively collected data from patients who underwent EVT for stroke at the First Hospital of Shanxi Medical University from January 2020 to June 2025. Inclusion criteria were: (1) patients who received EVT within 24 h of stroke onset; (2) age ≥18 years; (3) stroke caused by severe stenosis or occlusion of the large arteries, confirmed by digital subtraction angiography (DSA). Exclusion criteria were: (1) Posterior circulation stroke; (2) absence of post-operative head CT scans; (3) Loss to 90-day missing outcome data.

### Baseline data collection

This study collected demographic, clinical, and laboratory characteristics, as well as imaging features of the patients enrolled. Demographic data included age, gender, height and weight. Clinical features included smoking history, admission blood pressure, Preoperative National Institutes of Health Stroke Scale (NIHSS), and the administration of intravenous thrombolysis. Medical history included hypertension, diabetes, History of Ischemic Stroke, History of Intracranial hemorrhage, and coronary artery disease. Laboratory data included triglycerides (TG), total cholesterol (TC), low-density lipoprotein cholesterol (LDL-C), high-density lipoprotein cholesterol (HDL-C), Red blood cell count (RBC), neutrophils, platelet count (PLT), urea, creatinine, Hemoglobin A1c (HbA1c), homocysteine, albumin, and fasting blood glucose. Surgical characteristics included onset-to-puncture time (OPT), the number of EVTs, and number of patients with preoperative TICI grade >0 and postoperative TICI grade <2b. Imaging features included infarct volume, cortical infarction, and subcortical infarction. Blood samples were collected in a fasting state within 24 h after admission and analyzed by laboratory professionals. Surgical characteristics were extracted from the operative records. The number of EVTs included thrombectomy, stent retrieval, balloon angioplasty, stent implantation, and intra-arterial thrombolysis.

### Imaging analysis

All patients underwent head CT within 24 h post-EVT and were re-examined 5–7 days later to differentiate HT from contrast agent retention. The feature of contrast retention is that it typically disappears in follow-up CT scans. HT was classified based on the European Cooperative Acute Stroke Study II (ECASS II) criteria (14), dividing HT into two major subtypes: hemorrhagic infarction (HI) and parenchymal hematoma (PH). HI and PH were further divided into HI1 (small punctate hemorrhage at the infarct margin), HI2 (fused punctate hemorrhages within the infarct area without mass effect), PH1 (blood clot in ≤30% of the infarct area with mild mass effect), and PH2 (>30% of the infarct area with blood clot and significant mass effect).

The ischemic infarct pattern was categorized into: (1) subcortical regions (whether or not cortical areas are involved); (2) only cortical regions. Subcortical infarction areas included the caudate nucleus, putamen, internal capsule, and thalamus.

Infarct volume was calculated using the open-source software 3D-Slicer,^1^ manually segmenting the infarct area in each slice and measuring the infarct area on each slice. The infarct volume for each slice was calculated by multiplying the infarct area by the slice thickness. The total infarct volume was the sum of the volumes from all slices. CT images from postoperative days 3 to 7 were preferred for infarct evaluation, as the boundaries of infarcts are most clearly defined during this period. When HT occurred, the total infarct volume was first calculated, followed by the calculation of the hemorrhagic area volume. The non-hemorrhagic volume was derived by subtracting the hemorrhagic area volume from the total infarct volume. Hemorrhages located outside the infarct region (remote intracranial hemorrhage) were not included in infarct volume adjustment. Preoperative CT or MRI scans were reviewed to identify pre-existing encephalomalacia. These encephalomalacia were excluded from infarct volume measurement.

### Clinical outcomes

Clinical outcome measures in this study, in addition to HT and its subtypes, included early neurological deterioration (END), mortality at 7 days and 90 days post-admission, and neurological function scores at multiple time points. The 90-day mortality rate was obtained through outpatient visits and follow-up phone calls. Neurological function was assessed using the NIHSS, with NIHSS scores recorded at 24, 48, and 72 h after admission, as well as the change in NIHSS scores from pre-treatment to 7 days and from 24 h to 7 days post-treatment. END was defined as a worsening of neurological deficits within 24 h post-surgery, with an increase in the NIHSS score ≥4 points from baseline (15).

### Statistical analysis

The study population was divided into two groups based on infarct volume: small infarct volume group (0–15 mL) and large infarct volume group (≥70 mL), with separate analyses conducted for each group. These grouping thresholds were determined according to the infarct volume distribution characteristics observed in our study population. Continuous variables were expressed as mean ± standard deviation (SD) or median and interquartile range (IQR), while categorical variables were presented as counts (*n*) and percentages (%). Differences between groups were compared using appropriate statistical tests based on variable types and distribution characteristics: Student’s *t*-test or Mann–Whitney *U* test for continuous variables, and chi-square or Fisher’s exact test for categorical variables. Univariate and multivariate logistic regression analyses were conducted for the small and large infarct volume groups, using the presence of HT (and PH as a more severe HT type) as the dependent variable. The odds ratio (OR) and 95% confidence intervals (CI) were reported. Variables that were statistically significant in univariate analysis were included in the multivariate logistic regression model to identify independent predictors. To explore the relationship between different HT subtypes and END, comparisons were made within the small and large infarct volume groups using chi-square or Fisher’s exact test. Due to the small number of deaths in the small infarct group, the relationship between different HT subtypes and mortality was only analyzed in the large infarct volume group and total patients, using the same statistical method. Statistical significance was set at a two-sided *p*-value ≤ 0.05. All statistical analyses were performed using R version 4.1.3 and IBM SPSS Statistics 27.

## Results

### Study population

**Figure 1:**
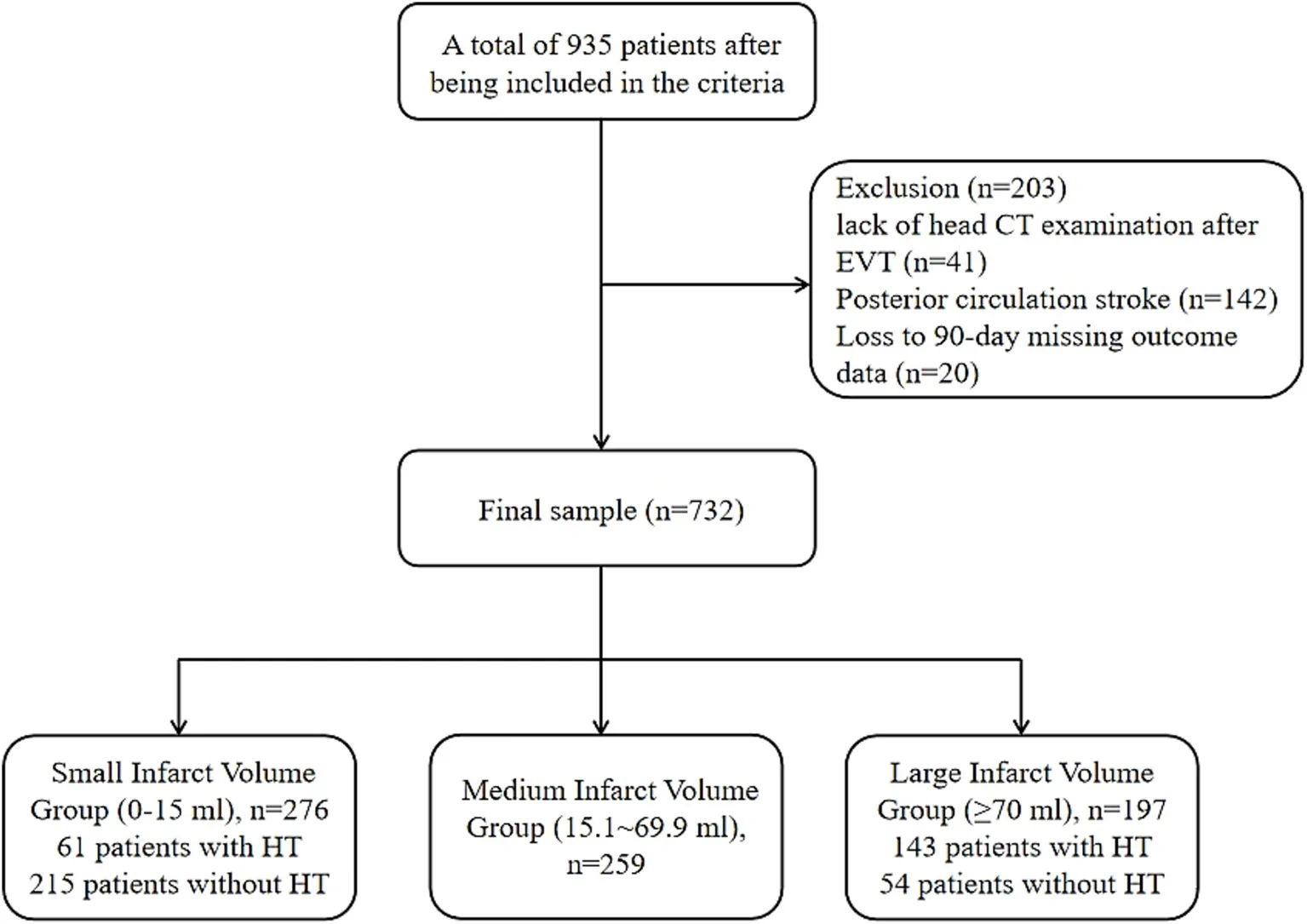
Flow chart of participants’ selection.

A total of 732 patients met the inclusion criteria (Figure 1), including 276 in the small infarct volume group (0–15 mL) and 197 in the large infarct volume group (≥70 mL). Baseline characteristics are summarized in Supplementary Table S1.

### Infarct volume distribution and HT on follow-up CT

Supplementary Figure S1 shows that the majority of patients had infarct volumes concentrated in the lower range, with a rapid decrease in case numbers as infarct volume increased. Supplementary Figure S2 shows the CT manifestations of HT subtypes in infarcts of small and large volumes.

### Risk factors for HT

In the small infarct volume group, patients who developed HT were generally older and had higher SBP, greater preoperative NIHSS scores, lower platelet counts and others than those without HT. They also underwent a higher number of EVT attempts and had lower triglyceride levels. In contrast, in the large infarct volume group, HT was mainly associated with more severe baseline neurological deficits, higher albumin levels, and more frequent the number of EVTs (Table 1). While the median NIHSS scores between groups may appear numerically close, statistical significance was determined based on the overall distribution of scores rather than solely on median differences.

**Table 1:** HT factors in patients with infarct volumes.

	Small infarct volume group (0–15 mL), *n* = 276			Large infarct volume group (≥70 mL), *n* = 197		
	without HT (*n* = 215)	HT (*n* = 61)	*p*-value	without HT (*n* = 54)	HT (*n* = 143)	*p*-value
Demographic characteristics						
Age mean ± SD, years	64.31 ± 12.35	68.11 ± 12.25	**0.034**	65.24 ± 12.44	65.77 ± 12.43	0.790
Male *n* (%)	153 (71.16)	42 (68.86)	0.728	35 (64.81)	100 (69.93)	0.493
Height mean ± SD, cm	167.72 ± 7.53	167.57 ± 7.93	0.898	166.69 ± 7.91	167.26 ± 7.65	0.642
Weight mean ± SD, Kg	69.33 ± 13.24	67.36 ± 12.49	0.301	70.42 ± 12.86	69.97 ± 10.59	0.803
Clinical characteristics						
Smoking *n* (%)	95 (44.19)	32 (52.46)	0.253	24 (44.44)	66 (46.15)	0.830
SBP mean ± SD, mmHg	138.69 ± 19.03	147.20 ± 24.33	**0.004**	139.91 ± 23.05	141.1 ± 22.50	0.744
DBP mean ± SD, mmHg	81.84 ± 13.21	83.03 ± 14.58	0.543	80.17 ± 14.23	80.29 ± 12.71	0.978
Preoperative NIHSS median (IQR)	9 (6–12)	10 (8–13)	**0.010**	13 (9–15)	15 (11–17)	**0.018**
Intravenous thrombolysis *n* (%)	50 (23.26)	18 (29.51)	0.324	16 (29.62)	46 (32.17)	0.731
Medical history						
Hypertension *n* (%)	117 (54.42)	37 (60.66)	0.385	39 (72.22)	97 (67.83)	0.550
Diabetes *n* (%)	52 (24.19)	15 (24.59)	0.948	16 (29.62)	43 (30.07)	0.952
History of ischemic Stroke *n* (%)	57 (26.51)	8 (13.11)	**0.022**	16 (29.62)	30 (20.98)	0.208
History of intracranial hemorrhage *n* (%)	5 (2.33)	2 (3.28)	0.652	1 (1.82)	0	–
Atrial fibrillation *n* (%)	42 (19.53)	11 (18.03)	0.791	12 (22.22)	37 (25.87)	0.594
Coronary heart disease *n* (%)	28 (13.02)	7 (11.48)	0.746	9 (16.66)	21 (14.69)	0.732
Laboratory characteristics						
TC mean ± SD, mmol/L	4.16 ± 0.98	4.21 ± 1.24	0.332	4.20 ± 1.28	4.18 ± 1.08	0.936
TG mean ± SD, mmol/L	1.51 ± 0.97	1.22 ± 0.48	**0.002**	1.35 ± 0.66	1.40 ± 0.81	0.696
LDL-C mean ± SD, mmol/L	2.66 ± 0.75	2.56 ± 0.89	0.406	2.66 ± 0.91	2.65 ± 0.81	0.899
HDL-C mean ± SD, mmol/L	1.07 ± 0.23	1.14 ± 0.25	**0.035**	1.08 ± 0.27	1.09 ± 0.25	0.746
RBC mean ± SD, 10^12^/L	4.52 ± 0.58	4.43 ± 0.50	0.285	4.47 ± 0.66	4.47 ± 0.62	0.981
Neutrophil mean ± SD, 10^9^/L	5.99 ± 2.91	6.31 ± 2.31	0.438	7.26 ± 3.08	7.92 ± 3.36	0.211
PLT mean ± SD, 10^9^/L	220.97 ± 66.06	199.20 ± 60.52	**0.021**	226.07 ± 67.11	212.57 ± 75.68	0.251
Urea mean ± SD, mmol/L	5.74 ± 1.99	5.78 ± 2.21	0.903	5.81 ± 2.16	5.85 ± 2.64	0.922
Creatinine mean ± SD, μmol/L	70.35 ± 16.96	71.40 ± 33.82	0.816	71.18 ± 23.52	72.16 ± 31.46	0.836
HbA1c mean ± SD, %	6.41 ± 1.46	6.55 ± 1.69	0.528	6.71 ± 1.63	6.72 ± 1.57	0.926
Homocysteine mean ± SD, μmol/L	19.71 ± 13.74	18.88 ± 11.91	0.671	18.83 ± 11.95	20.50 ± 15.51	0.478
Albumin mean ± SD, g/L	39.52 ± 3.87	38.83 ± 4.36	0.229	37.65 ± 5.17	39.32 ± 4.41	**0.025**
Fasting blood sugar mean ± SD, mmol/L	7.05 ± 2.15	7.40 ± 2.38	0.280	7.98 ± 2.55	8.27 ± 2.60	0.492
Surgical characteristics						
OPT median (IQR), min	460 (300–720)	560 (350–840)	0.121	425 (223–683)	420 (300–620)	0.669
Preoperative TICI > 0, *n* (%)	24 (11.16)	1 (1.64)	**0.021**	2 (3.64)	0	–
Postoperative TICI < 2b, *n* (%)	7 (3.26)	1 (1.64)	0.690	5 (9.09)	24 (16.90)	0.167
The number of EVTs median (IQR)	2 (1–3)	2 (1–3)	**0.005**	2 (1–3)	3 (2–3)	**0.031**
Imaging characteristics						
Infarct volume median (IQR), ml	6 (3–10)	10 (7–12)	**<0.001**	157 (90–238)	154 (97–232)	0.859
Cortical infarction *n* (%)	101 (46.98)	14 (22.95)	**<0.001**	7 (12.96)	12 (8.39)	0.345
Subcortical infarction *n* (%)	114 (53.02)	47 (77.05)		47 (87.04)	131 (91.61)	

IQR, interquartile range; SD, standard deviation; HT, hemorrhagic transformation; SBP, systolic blood pressure; DBP, diastolic blood pressure; NIHSS, national institutes of health stroke scale; TG, triglycerides; TC, total cholesterol; LDL-C, low-density lipoprotein cholesterol; HDL-C, high-density lipoprotein cholesterol; RBC, red blood cells; PLT, platelet; HbA1c, hemoglobin A1c; EVT, endovascular treatment; OPT: onset to puncture time.

### Relationship between infarct volume and HT incidence

Across the overall cohort, the incidence and severity of HT increased with infarct volume. The proportion of parenchymal hematomas (PH1 and PH2) rose significantly as infarct size grew (Supplementary Table S2).

### Univariate and multivariate analysis

Univariate analysis found (Supplementary Table S3) that in the small infarct volume group, HT occurrence was significantly associated with higher SBP, higher NIHSS score, lower TG, lower platelet count, cortical infarction, and others (all *p* < 0.05). Multivariate analysis confirmed systolic blood pressure (OR = 1.02, 95% CI 1.00–1.07, *p* = 0.020), TG (OR = 0.51, 95% CI 0.30–0.89, *p* = 0.017), and EVT frequency (OR = 1.74, 95% CI 1.24–2.45, *p* = 0.002) as independent predictors.

In the large infarct volume group, NIHSS score (OR 1.10, 95% CI 1.02–1.18, *p* = 0.010), albumin levels (OR = 1.10, 95% CI 1.0–1.18, *p* = 0.010), and EVT frequency (OR = 1.51, 95% CI 1.11–2.05, *p* = 0.009) were identified as independent predictors.

### HT subtypes and NIHSS changes

In the small infarct volume group, patients with PH2-type hemorrhage had significantly higher NIHSS scores at 24 h, 48 h, and 7 days post-admission compared to the without HT group (all *p* < 0.05), indicating more severe neurological deficits. Differences between HI and PH1 subtypes were smaller, with some time points showing no significant differences.

In the large infarct volume group, patients with PH2 hemorrhage exhibited significantly higher NIHSS scores at all evaluated time points compared with both non-HT patients (all *p* < 0.05). Furthermore, NIHSS scores in the PH2 subgroup increased between 24 h and 7 days, indicating progressive neurological deterioration and poorer recovery (Table 2).

**Table 2:** The impact of HT on NIHSS change in patients with different infarct volumes.

Small infarct volume group (0–15 ml), *n* = 276							
	Without HT	HI (*n* = 36)	*p*-value	PH1 (*n* = 19)	*p*-value	PH2 (*n* = 6)	*p*-value
24-h NIHSS (IQR)	6 (6 to 8)	7 (4 to 10)	**0.046**	8 (4 to 10)	0.121	12 (8 to 17)	**0.009**
48-h NIHSS (IQR)	4 (2 to 7)	6 (4 to 8)	**0.008**	6 (3 to 9)	**0.034**	12 (7 to 26)	**0.006**
7-day NIHSS (IQR)	3 (1 to 5)	4 (3 to 6)	**0.006**	4 (3 to 6)	**0.022**	10 (5 to 14)	**0.001**
Change in NIHSS score from 24 h to 7 days (IQR)	−2 (−4 to −1)	−2 (−4 to −1)	0.411	−2 (−5 to −1)	0.570	−3 (−6 to 0)	0.824
Change in NIHSS score from preoperative to 7 days (IQR)	−5 (−8 to −2)	−6 (−9 to −4)	0.291	−6 (−9 to −2)	0.578	−5 (−7 to 1)	0.524

Large infarct volume group (≥70 ml), *n* = 197							
	Without HT	HI (*n* = 36)	*p*-value	PH1 (*n* = 53)	*p*-value	PH2 (*n* = 54)	*p*-value
24-h NIHSS (IQR)	16 (11 to 21)	18 (10 to 25)	0.550	18 (12 to 24)	0.156	24 (16 to 30)	**<0.001**
48-h NIHSS (IQR)	14 (11 to 22)	17 (10 to 27)	0.569	16 (11 to 25)	0.412	27 (18 to 32)	**<0.001**
7-day NIHSS (IQR)	12 (8 to 16)	12 (8 to 25)	0.615	13 (8 to 19)	0.344	26 (14 to 42)	**<0.001**
Change in NIHSS score from 24 h to 7 days (IQR)	−2 (−6 to 1)	−2 (−7 to 5)	0.846	−3 (−8 to 1)	0.514	3 (−4 to 10)	**0.011**
Change in NIHSS score from preoperative to 7 days (IQR)	−1 (−4 to 5)	−2 (−5 to 9)	0.769	−1 (−5 to 6)	0.970	9 (0 to 22)	**<0.001**

### HT subtypes and END

**Figure 2:**
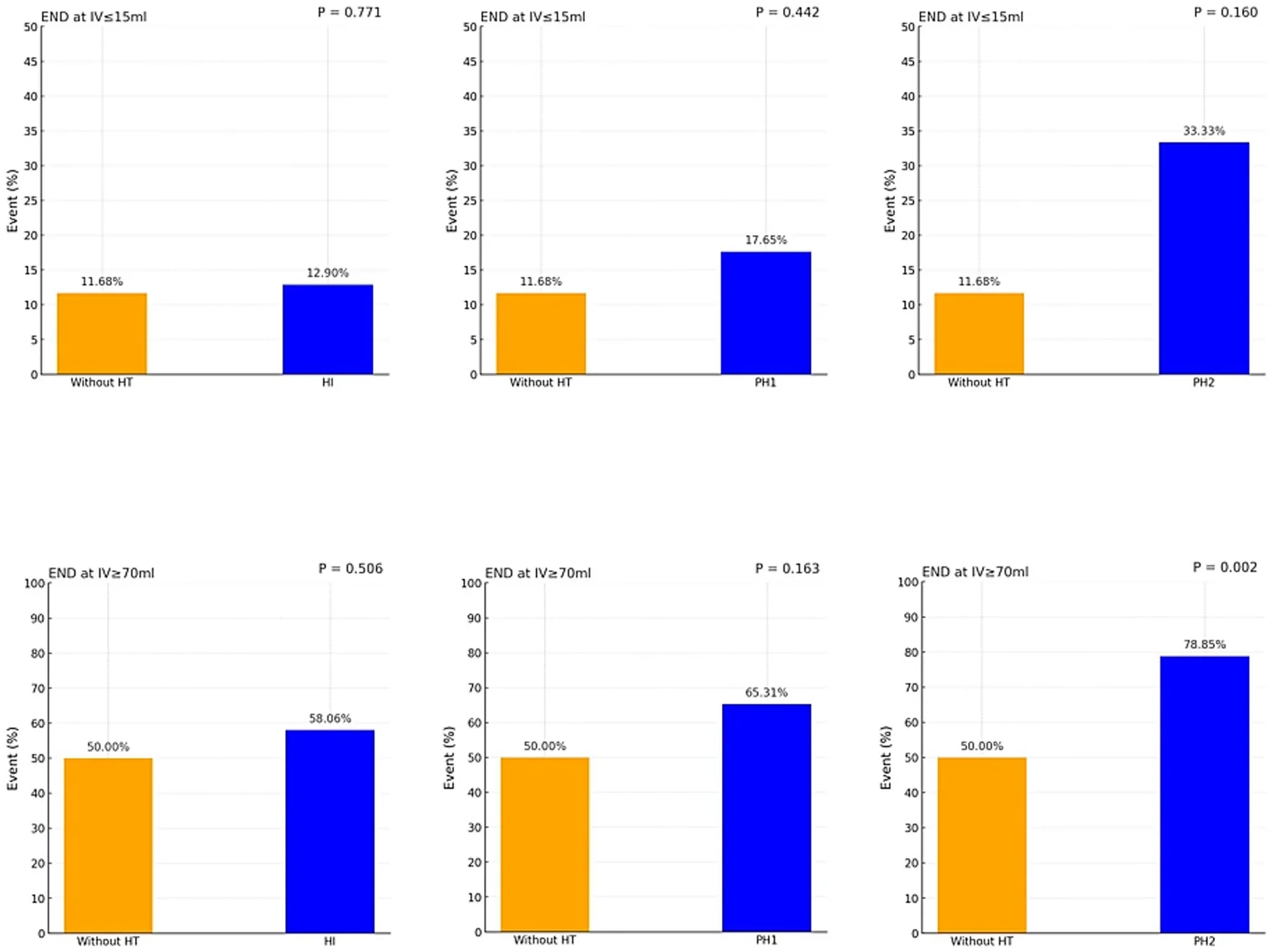
Comparison of END at infarction volumes in Patients subtupes of HT and without HT.

In the large infarct volume group, after excluding patients with bleeding occurring beyond 24 h post-admission, the incidence of END was significantly higher in PH2-type patients (78.85% vs. 50.00%, *p* = 0.002), whereas PH1-type (65.31%, *p* = 0.163) and HI-type (58.06%, *p* = 0.506) showed no significant differences compared to the without HT group. No significant differences in END were observed for any subtypes in the small infarct volume group (Figure 2).

### HT subtypes and mortality

**Figure 3:**
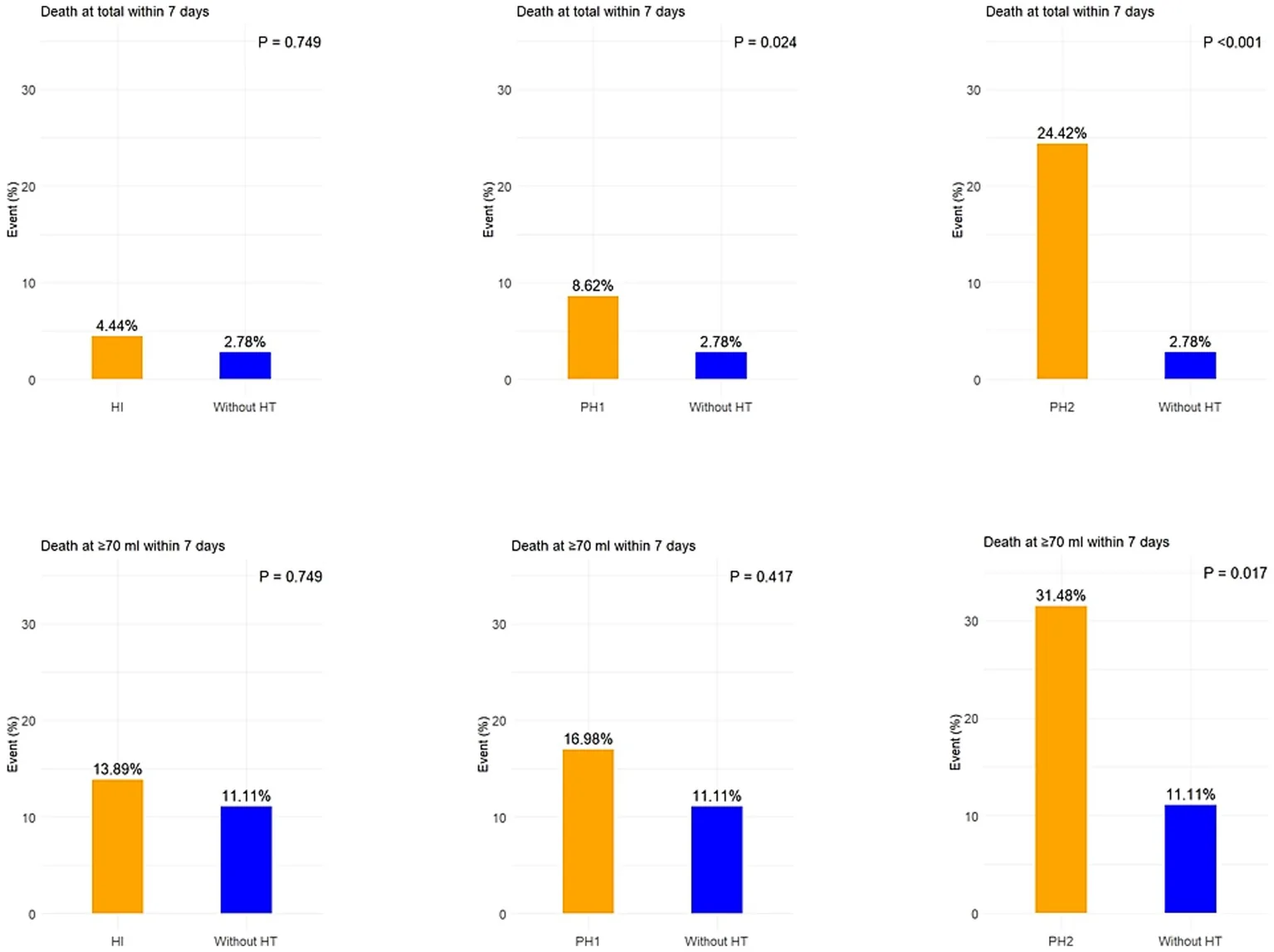
Comparison of 7-day mortality between HT subtypes and non-HT in patients with total and ≥70 mL.

In the overall population, the 7-day mortality rate of patients with PH2 hemorrhage was significantly higher than that of patients without HT (24.42% vs. 2.78%, *p* < 0.001). The mortality rate for PH1 hemorrhage patients was also slightly higher (8.62% vs. 2.78%, *p* = 0.024), while there was no significant difference for HI hemorrhage patients (*p* = 0.749). In the large infarct volume group, the 7-day mortality rate for PH2 hemorrhage patients was similarly significantly increased (31.48% vs. 11.11%, *p* = 0.017), while no significant difference was observed for PH1 (16.98%, *p* = 0.417) or HI (*p* = 0.749) hemorrhage patients compared to those without HT (Figure 3).

**Figure 4:**
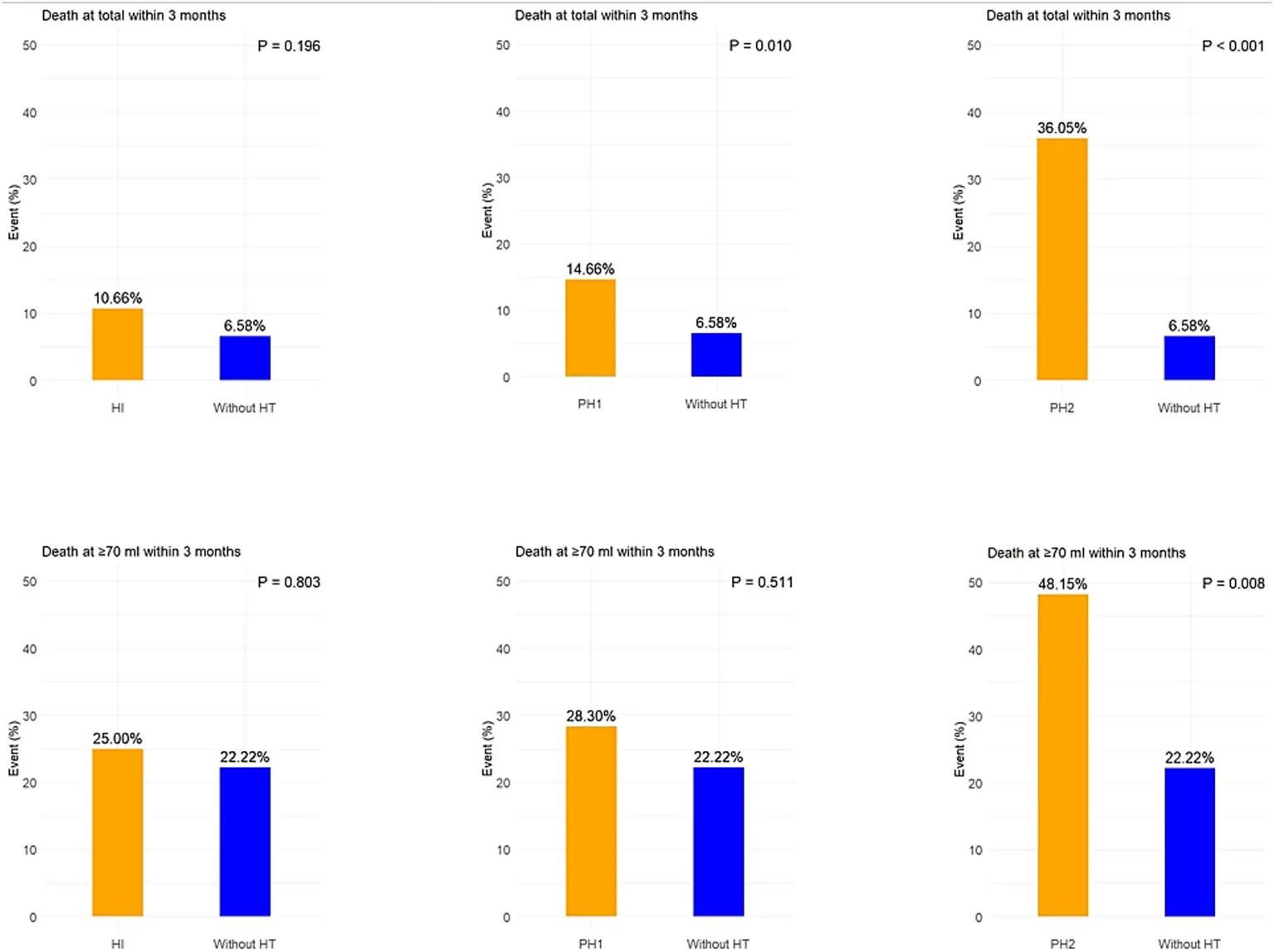
Comparison of 3-month mortality between HT subtypes and non-HT in patients with total and ≥70 mL.

At 3 months follow-up, the overall trend remained consistent with the short-term results. In the overall population, the 3-month mortality rate for PH2 hemorrhage patients was significantly higher than for those without HT (36.05% vs. 6.58%, *p* < 0.001), and PH1 hemorrhage patients also showed higher mortality (14.66% vs. 6.58%, *p* = 0.010), while there was no significant difference for HI hemorrhage patients (*p* = 0.196). In the large infarct volume group (≥70 mL), only PH2 hemorrhage patients had a significantly higher 3-month mortality rate (48.15% vs. 22.22%, *p* = 0.008), while no significant difference was found for PH1 or HI hemorrhage patients (*p* > 0.5) (Figure 4).

## Discussion

The results of this study indicate that, after EVT for ischemic stroke, patients with large infarct volumes (≥70 mL) are more likely to experience HT than those with small infarct volumes (0–15 mL), and the degree of hemorrhage is more severe. In the small infarct volume group, the occurrence of HT was closely associated with factors such as advanced age, high systolic blood pressure, pre-treatment NIHSS score, and other factors. In contrast, in the large infarct volume group, pre-treatment NIHSS score, elevated albumin levels, and EVT frequency were influencing factors of HT.

Furthermore, PH2 hemorrhage was associated with more severe neurological deficits, higher rates of END, and increased short-term and long-term mortality. This suggests that PH2 hemorrhage is the most adverse prognostic subtype, particularly in patients with large infarcts, where its impact on outcomes is more pronounced.

Infarct volume serves as an important indicator of the extent of brain tissue injury in ischemic stroke, reflecting both the degree of necrosis and the loss of perfusion. Generally, larger infarct volumes are associated with greater clinical severity (16). Previous studies have reported that infarct volume measurement is useful for early prognostic assessment and is strongly associated with functional outcomes after ischemic stroke (17). HT results from ischemia-induced vascular disruption, permitting blood extravasation into the infarcted parenchyma and leading to secondary neuronal injury. Traditionally, mild or “asymptomatic” hemorrhagic transformations were considered clinically insignificant. However, research indicates that even asymptomatic HI or subarachnoid hemorrhage (SAH) is associated with poorer functional recovery at 90 days in patients undergoing EVT for acute ischemic stroke (8, 18).

In addition to infarct volume, factors such as older age, hypertension, and diabetes increase the risk of HT due to impaired vascular elasticity, which makes blood vessel walls more fragile. Other factors, such as atrial fibrillation and platelet levels upon admission, can also increase the risk of HT (19). However, these factors are often uncontrollable. Furthermore, anticoagulants, antiplatelet drugs, and thrombolysis can increase the bleeding risk. A meta-analysis of randomized trials involving 2,313 patients reported a higher incidence of any intracranial hemorrhage in those receiving both thrombolysis and EVT compared with EVT alone (36% vs. 32%), underscoring the complex interplay of treatment-related and patient-specific factors (20).

Additionally, the location of brain infarction influences the risk of HT. Infarctions in deep brain structures, such as the basal ganglia and internal capsule, carry a higher risk of vascular damage due to the larger and more complex vessels in these areas, as well as drastic blood flow changes during reperfusion, which can easily lead to vessel rupture. On the other hand, cortical infarctions, while more stable in terms of blood flow dynamics, may still experience HT if the infarct volume is large, due to local blood flow fluctuations or mechanical damage to blood vessels (21). Furthermore, the operator’s choice of surgical approach and frequency of treatment may be key factors influencing the risk of HT. Mechanical damage to blood vessels (such as punctures or vessel wall injury) and hemodynamic changes during EVT can increase the risk of HT. These factors may be more prominent in patients with frequent and challenging procedures (22).

HT following stroke may involve pathological changes, including the sustained activation of oxidative stress and inflammatory cascades. These processes promote neuroinflammation, disrupt the blood–brain barrier (BBB), and increase vascular permeability, which in turn allows white blood cells and plasma proteins to infiltrate brain tissue, exacerbating brain edema and triggering HT (23–25). Matrix metalloproteinase-9 (MMP-9) has been found to play a significant role in degrading key BBB proteins, promoting vessel rupture, and facilitating blood leakage (26). Additionally, reperfusion injury is considered one of the key mechanisms. While rapid reperfusion is the goal of EVT, reperfusion itself paradoxically exacerbates tissue damage (27). Moreover, we observed that, in patients with small infarcts, HT was associated with multiple systemic and procedural factors, whereas fewer independent predictors were identified in patients with large infarcts. This may reflect fundamental pathophysiological differences between these groups. Consequently, the development of HT in patients with smaller infarcts may depend more on clinical or hemodynamic factors, whereas in those with large infarcts, intrinsic tissue vulnerability predominates. The consistent association between the number of EVT passes and HT across groups further emphasizes the procedural contribution to this complication. Therefore, special attention should be paid to this high-risk population when planning and performing treatment to reduce the incidence of HT.

The overall incidence of HT in this cohort was approximately 46%, which aligns with prior studies reporting rates between 40 and 50% among EVT-treated patients (21). Moreover, the median infarct volume in this study was 27 mL, which is similar to the 24.9 mL reported in previous studies of EVT patients, and the distribution of infarct volumes was also consistent (28). The median infarct volume (27 mL) was also comparable to earlier series, supporting the representativeness of this cohort (29). Notably, in the small infarct volume group, patients with HT had consistently higher NIHSS scores at various stages post-admission compared to those without HT, indicating that HT, regardless of type, is associated with worse outcomes compared to without HT.

Although atrial fibrillation has been widely reported as a risk factor for HT, it was not identified as an independent predictor after stratification by infarct volume in our study. When analyzing the overall cohort without volume stratification, AF was associated with HT. This suggests that the effect of atrial fibrillation on HT risk may be partially mediated through larger infarct burden rather than acting as a direct independent predictor.

This study has several limitations. First, in calculating infarct volume, potential errors may have occurred due to manual measurement, and CT has limited spatial resolution for detecting small infarct areas. Although head MRI was used when the infarct range was unclear on CT, some patients lacked MRI data, which may have affected the accuracy of infarct volume estimation. Second, previous studies have indicated that infarct volume can expand during hospitalization, regardless of whether complete vascular recanalization is achieved. This phenomenon may have influenced the analysis of the relationship between infarct volume and short-term prognosis (30). Finally, the study’s regional and demographic limitations may affect the external validity and generalizability of the results.

## Conclusion

In patients with acute ischemic stroke, infarct volume is closely related to the occurrence and severity of HT. In the small infarct volume group, HT is primarily associated with older age, SBP, neurological deficits, low platelet count, subcortical infarction, and other factors. In the large infarct volume group, Influencing factors include preoperative neurological deficits, albumin levels, and EVT frequency. PH2-type hemorrhage significantly increases the risk of END and mortality, highlighting the need for special attention to this subtype in patients with large infarct volumes to optimize risk assessment and clinical decision-making.

## Data Availability

The original contributions presented in the study are included in the article/Supplementary material, further inquiries can be directed to the corresponding authors.

## Footnotes

1
https://download.slicer.org/
