# Correction: Analysis of discrepancies in hemorrhagic transformation and infarct volume in ischemic stroke patients undergoing endovascular treatment

**DOI:** 10.3389/fneur.2026.1978162

**Published:** 2026-10-06

**Authors:** Jianqiang Hu, Shuyu Ma, Jiawei Zhang, Kefangyuan Zheng, Mingqing Cheng, Xin Miao, Jiarui Bao, Donghua Xian, Yalan Fang, Jin Zhang

**Affiliations:** 1 Department of Neurology, Second Hospital of Shanxi Medical University, Taiyuan, Shanxi, China; 2 Clinical College, Shanxi Medical University, Taiyuan, Shanxi, China; 3 Department of Neurology, First Hospital of Shanxi Medical University, Taiyuan, Shanxi, China

**Keywords:** endovascular treatment, hemorrhagic transformation, infarct volume, ischemic stroke, neuralfunctional outcomes

Jianqiang Hu^1†^, Shuyu Ma^2†^, Jiawei Zhang^2^, Kefangyuan Zheng^2^, Mingqing Cheng^2^, Xin Miao^2^, Jiarui Bao^2^, Donghua Xian^2^, Yalan Fang^1*^ and Jin Zhang^3*^

^1^Department of Neurology, Second Hospital of Shanxi Medical University, Taiyuan, Shanxi, China,

^2^Clinical College, Shanxi Medical University, Taiyuan, Shanxi, China,

^3^Department of Neurology, First Hospital of Shanxi Medical University, Taiyuan, Shanxi, China

The affiliation for author Jianqiang Hu was incorrectly listed in the published article. The affiliation was corrected from “Clinical College, Shanxi Medical University, Taiyuan, Shanxi, China” to “Department of Neurology, Second Hospital of Shanxi Medical University, Taiyuan, Shanxi, China”. All other authors and their affiliations remain unchanged however the original first two affiliations have swapped places.

The original version of this article has been updated.

