# Supplementary material for: Analysis of discrepancies in hemorrhagic transformation and infarct volume in ischemic stroke patients undergoing endovascular treatment
Source: Front Neurol. 2026 Mar 6;17:1783768. doi: 10.3389/fneur.2026.1783768 (PMC13002435; doi:10.3389/fneur.2026.1783768)
Supplement: Supplementary file 1 [file Table_1.docx]

**Table S1.** Baseline Characteristics of All Patients

|  | All Patients (n=732) |
| --- | --- |
| Demographic characteristics | |
| Age mean ± SD, years | 65.45 ± 12.75 |
| Male n (%) | 485 (66.26) |
| Height mean ± SD, cm | 167.02 ± 7.64 |
| Weight mean ± SD, Kg | 68.47 ± 12.47 |
| Clinical characteristics | |
| Smoking n (%) | 324 (44.26) |
| SBP mean ± SD, mmHg | 141.11 ± 21.63 |
| DBP mean ± SD, mmHg | 81.71 ± 14.03 |
| Preoperative NIHSS median (IQR) | 11 (8~15) |
| Intravenous Thrombolysis n (%) | 190 (25.96) |
| TOAST Classification | |
| Large Artery Atherosclerotic Stroke n (%) | 515 (70.36) |
| Cardioembolic Stroke n (%) | 184 (25.14) |
| Other Determined Etiology n (%) | 12 (1.63) |
| Cryptogenic Stroke n (%) | 21 (2.67) |
| Medical History | |
| Hypertension n (%) | 440 (60.11) |
| Diabetes n (%) | 191 (26.09) |
| History of Ischemic Stroke n (%) | 164 (22.40) |
| History of Intracranial hemorrhage n (%) | 13 (1.78) |
| Atrial Fibrillation n (%) | 177 (24.18) |
| Coronary Heart Disease n (%) | 102 (13.93) |
| Laboratory Characteristics | |
| TC mean ± SD, mmol/L | 4.18 ± 1.05 |
| TG mean ± SD, mmol/L | 1.40 ± 0.84 |
| LDL-C mean ± SD, mmol/L | 1.10 ± 0.25 |
| HDL-C mean ± SD, mmol/L | 2.64 ± 0.78 |
| RBC mean ± SD, 10^12/L | 4.46 ± 0.62 |
| Neutrophil mean ± SD, 10^9/L | 6.74 ± 3.00 |
| PLT mean ± SD, 10^9/L | 214.05 ± 67.38 |
| Urea mean ± SD, mmol/L | 5.88 ± 2.79 |
| Creatinine mean ± SD, μmol/L | 71.55 ± 27.46 |
| HbA1c mean ± SD, % | 6.50 ± 1.52 |
| Homocysteine mean ± SD, μmol/L | 20.34 ± 15.62 |
| Albumin mean ± SD, g/L | 39.08 ± 4.35 |
| Fasting blood sugar mean ± SD, mmol/L | 7.40 ± 2.32 |
| Surgical Characteristics | |
| OPT median (IQR), min | 460 (300~720) |
| Preoperative TICI> 0, n (%) | 42 (5.74) |
| Postoperative TICI < 2b, n (%) | 50 (6.83) |
| the number of EVTs median (IQR) | 1 (1~2) |
| Imaging Characteristics | |
| Infarct Volume median (IQR), ml | 27 (9~78) |
| Cortical Infarction n (%) | 208 (28.42) |
| Subcortical infarction only n (%) | 524 (71.58) |

**Abbreviations:** IQR, interquartile range; SD, standard deviation; SBP, systolic blood pressure; DBP, diastolic blood pressure; NIHSS, National Institutes of Health Stroke Scale; TOAST Classification, Trial of ORG 10172 in Acute Stroke Treatment classification; TG, Triglycerides; TC, Total Cholesterol; LDL-C, Low-Density Lipoprotein Cholesterol; HDL-C, High-Density Lipoprotein Cholesterol; RBC, Red blood cells; PLT, Platelet; HbA1c, Hemoglobin A1c; EVT, Endovascular Treatment; OPT: Onset to Puncture Time

**Table S2** HT and Subtupes in each infarct volume groups

|  | Small Infarct Volume Group (0-15 ml), n=276 | Medium Infarct Volume Group (15.1-69.9 ml), n=259 | Large Infarct Volume Group (≥70 ml), n=197 | p-value |
| --- | --- | --- | --- | --- |
| HT (%) | 61 (22.10) | 133 (51.35) | 143 (72.59) | **<0.001** |
| HI (%) | 36 (13.04) | 64 (24.71) | 36 (18.27) | **0.002** |
| PH1 (%) | 19 (6.88) | 44 (16.99) | 53 (26.90) | **<0.001** |
| PH2 (%) | 6 (2.17) | 26 (10.04) | 54 (27.41) | **<0.001** |

**Abbreviations:** HT, Hemorrhagic Transformation; HI, Hemorrhagic Infarction; PH, Parenchymal Hematoma

**Table S3.** Univariate and multivariate analyses of potential prognostic factors based on HT presence and infarct volumes

|  | Small Infarct Volume Group (0-15 ml), n=276 | | | Large Infarct Volume Group (≥70 ml), n=197 | | |
| --- | --- | --- | --- | --- | --- | --- |
|  | Univariate analysis | Multivariate analysis | | Univariate analysis | Multivariate analysis | |
|  | p-value | OR (95%CI) | p-value | p-value | OR (95%CI) | p-value |
| Demographic characteristics |  | | |  | | |
| Age mean (years) | **0.035** | 1.01 (0.98~1.04) | 0.491 | 0.789 | ---- | ---- |
| Male | 0.727 | ---- | ---- | 0.491 | ---- | ---- |
| Height mean (cm) | 0.897 | ---- | ---- | 0.640 | ---- | ---- |
| Weight (Kg) | 0.301 | ---- | ---- | 0.802 | ---- | ---- |
| Clinical characteristics |  | | |  | | |
| Smoking | 0.254 | ---- | ---- | 0.830 | ---- | ---- |
| SBP (mmHg) | **0.006** | 1.02 (1.00~1.07) | **0.020** | 0.739 | ---- | ---- |
| DBP (mmHg) | 0.541 | ---- | ---- | 0.978 | ---- | ---- |
| Preoperative NIHSS | **0.014** | 1.00 (0.92~1.07) | 0.897 | **0.016** | 1.10 (1.02~1.18) | **0.010** |
| Intravenous Thrombolysis | 0.318 | ---- | ---- | 0.732 | ---- | ---- |
| Medical History |  | | |  | | |
| Hypertension | 0.387 | ---- | ---- | 0.553 | ---- | ---- |
| Diabetes | 0.948 | ---- | ---- | 0.952 | ---- | ---- |
| History of Ischemic Stroke | **0.033** | 1.72 (0.69~4.28) | 0.224 | 0.203 | ---- | ---- |
| History of Intracranial hemorrhage | 0.678 | ---- | ---- | ---- | ---- | ---- |
| Atrial Fibrillation | 0.793 | ---- | ---- | 0.597 | ---- | ---- |
| Coronary Heart Disease | 0.749 | ---- | ---- | 0.730 | ---- | ---- |
| Laboratory Characteristics |  | | |  | | |
| TC (mmol/L) | 0.739 | ---- | ---- | 0.936 | ---- | ---- |
| TG (mmol/L) | **0.028** | 0.51 (0.30~0.89) | **0.017** | 0.695 | ---- | ---- |
| LDL-C (mmol/L) | 0.405 | ---- | ---- | 0.893 | ---- | ---- |
| HDL-C (mmol/L) | **0.037** | 4.25 (0.90~19.89) | 0.068 | 0.745 | ---- | ---- |
| RBC (10^12/L) | 0.285 | ---- | ---- | 0.981 | ---- | ---- |
| Neutrophil (10^9/L) | 0.437 | ---- | ---- | 0.211 | ---- | ---- |
| PLT (10^9/L) | **0.023** | 1.00 (0.99~1.00) | 0.146 | 0.252 | ---- | ---- |
| Urea (mmol/L) | 0.903 | ---- | ---- | 0.922 | ---- | ---- |
| Creatinine (μmol/L) | 0.741 | ---- | ---- | 0.835 | ---- | ---- |
| HbA1c (%) | 0.527 | ---- | ---- | 0.925 | ---- | ---- |
| Homocysteine (μmol/L) | 0.670 | ---- | ---- | 0.478 | ---- | ---- |
| Albumin (g/L) | 0.229 | ---- | ---- | **0.027** | 1.10 (1.02~1.18) | **0.010** |
| Fasting blood sugar (mmol/L) | 0.280 | ---- | ---- | 0.490 | ---- | ---- |
| Surgical Characteristics |  | | |  | | |
| OPT median (min) | 0.268 | ---- | ---- | 0.970 | ---- | ---- |
| Preoperative TICI> 0 | **0.050** | 0.25 (0.03~2.09) | 0.202 | ---- | ---- | ---- |
| Postoperative TICI < 2b | 0.515 | ---- | ---- | 0.190 | ---- | ---- |
| the number of EVTs median | **0.002** | 1.74 (1.24~2.45) | **0.002** | **0.027** | 1.51 (1.11~2.05) | **0.009** |
| Imaging Characteristics |  | | |  | | |
| Infarct Volume median (ml) | **<0.001** | 1.22 (1.11~1.33) | **<0.001** | 0.700 | ---- | ---- |
| Cortical Infarct | **0.001** | 2.51 (1.19~5.27) | **0.015** | 0.336 | ---- | ---- |
| Subcortical Infarct Area |  |  |  |  |  |  |


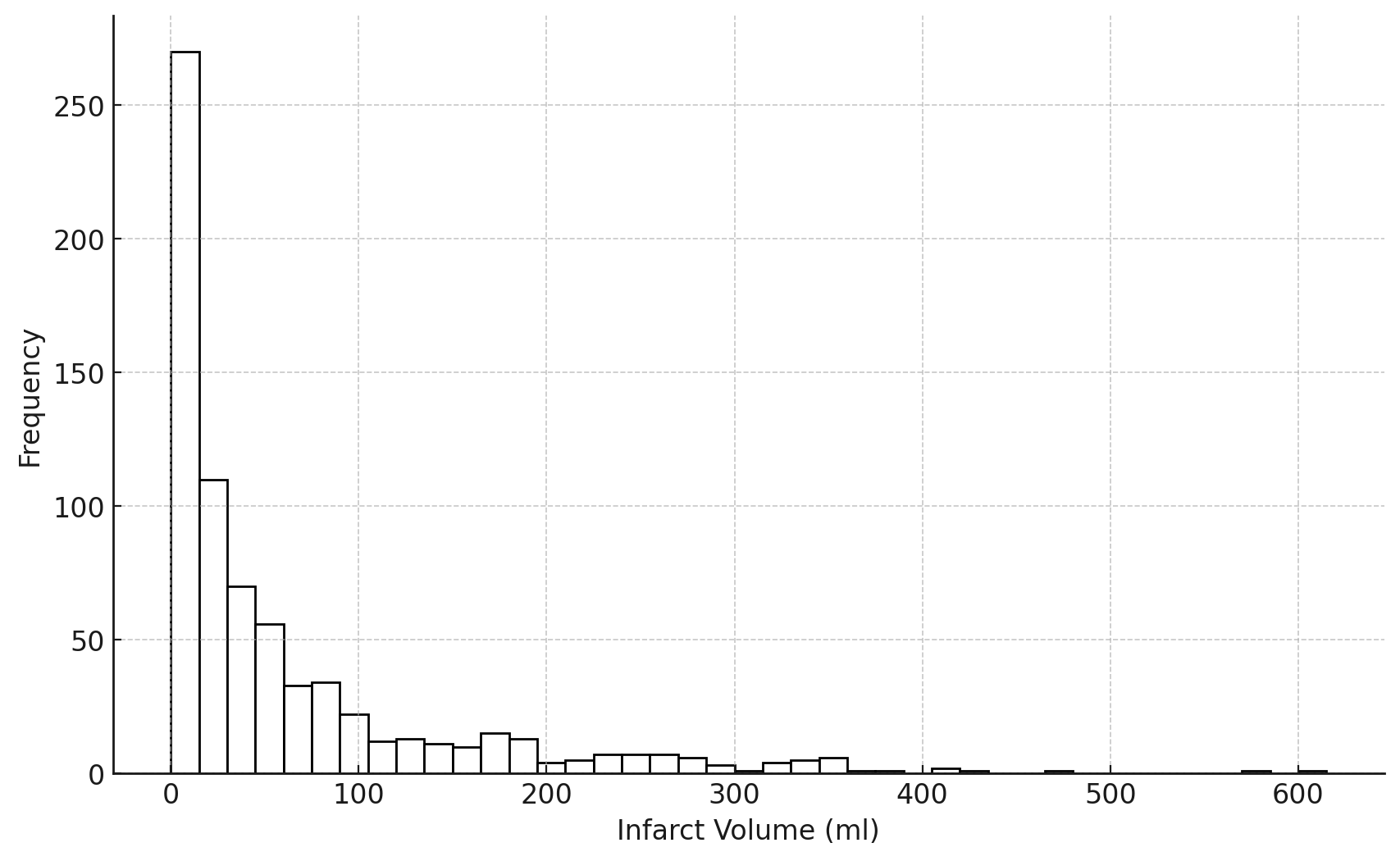


**Figure S1 Distribution of infarct volumes in the patient sample**

**
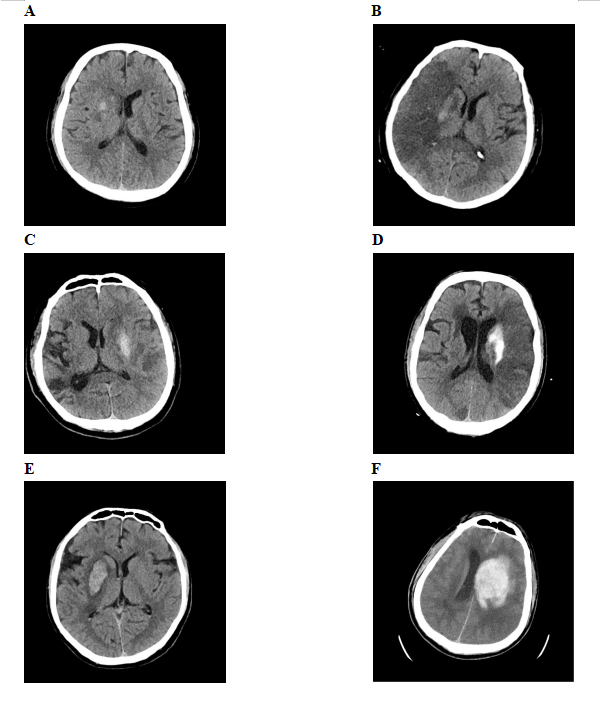
**

**Figure S2** The ACE group represents small infarct volume hemorrhagic transformations, including HI, PH1, and PH2 subtypes; the BDF group represents large infarct volume hemorrhagic transformations, encompassing HI, PH1, and PH2 subtypes.
